# Community stakeholder engagement during a vaccine demonstration project in Nigeria: lessons on implementation of the good participatory practice guidelines

**DOI:** 10.11604/pamj.2019.34.179.18458

**Published:** 2019-12-05

**Authors:** Morenike Oluwatoyin Folayan, Florita Durueke, Wika Gofwen, Godwin Godo-Odemijie, Chuks Okonkwo, Bali Nanmak, Sophia Osawe, Evaezi Okporoko, Alash’le Abimiku

**Affiliations:** 1 New HIV Vaccine and Microbicide Advocacy Society, Abuja, Nigeria; 2 Institute of Public Health, Obafemi Awolowo University, Ile-Ife, Nigeria; 3 Department of Child Dental Health, Obafemi Awolowo University, Ile-Ife, Nigeria; 4 Plateau State Human Virology Research Center, Jos Institute of Human Virology, Abuja, Nigeria; 5 Institute of Human Virology, School of Medicine, University of Maryland, Baltimore, USA

**Keywords:** Community stakeholder engagement, HIV vaccine, Nigeria

## Abstract

**Introduction:**

To report on the successes and challenges with implementing the good participatory practice guidelines for the Nigerian Canadian Collaboration on AIDS Vaccine (NICCAV) project.

**Methods:**

An open and close ended questionnaire was administered to 25 randomly selected community stakeholders on the project. The questions sought information on perception about the community entry, constitution and function of the community advisory board (CAB) and community based organization (CBO), media engagement process, and research literacy programmes. The quantitative and qualitative data were analysed and findings triangulated.

**Results:**

The project exceeded its targets on CBO engagement and community members reached. Stakeholders had significant improvement in knowledge about HIV vaccine research design and implementation (p=0.004). All respondents felt satisfied with the community entry, CAB constitution process, function and level of media engagement; 40% were satisfied with the financial support provided; 70% felt the community awareness and education coverage was satisfactory; and 40% raised concerns about the study site selection with implications for study participants' recruitment.

**Conclusion:**

The NICCAV community stakeholder engagement model produced satisfactory outcomes for both researchers and community stakeholders. The inclusion of an advocacy and monitoring plan enabled it to identify important challenges that were of ethical concerns for the study.

## Introduction

The field of HIV vaccine development was reinvigorated by the results of the ALVAC-HIV (vCP1521) (Sanofi Pasteur)/AIDSVAX B/E (Global Solutions for Infectious Diseases) trial that demonstrated 31.2% protection [1], and lower risk of HIV infection for the study population when compared with populations in other trials [2-6]. A number of studies with new products to boost the potency of this and others with new vaccine concepts are ongoing [7-9]. These studies require large numbers of volunteers required for the implementation of these studies. Nigeria could provide an ideal setting for conducting HIV vaccine trials since it has the second largest burden of HIV infection in Africa [10]. Also has the unique HIV subtype G, CRF02 A/G along with other recombinant forms that accounts for about 40% of its HIV infection [11]. The Nigerian Canadian Collaboration on AIDS Vaccine (NICCAV) project was developed to address this need. Its focus was to build local capacity to conduct HIV vaccine trials [12] through a four years “mock” clinical trial [13]. The project had multiple objectives one of which was to identify how best to mobilise and effectively engage stakeholders in a HIV vaccine research project, and to ensure the community benefits by having improved knowledge and understanding of HIV vaccine research and clinical trials. Community and stakeholder engagement was prioritised for this project because it was considered an ethical imperative [14,15] as it enhances the quality and outcome of research [15,16], facilitates social change emanating from individual and community empowerment in support of health and development [17] and enhances the science, the sense of joint ownership of a study by both researchers and research stakeholder [17]. In effect, it improves research implementation, procedures, and outcomes; and helps to build effective and sustainable research collaborations [18].

**Figure 1:**
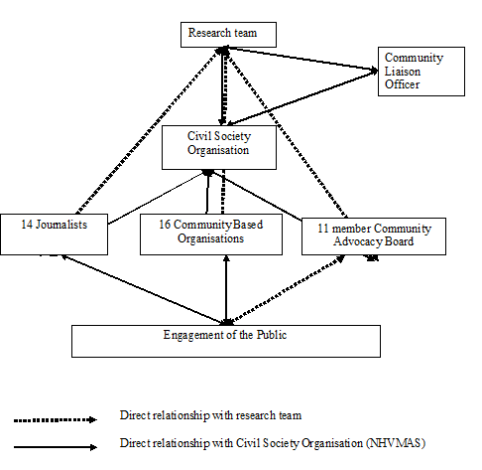
Stakeholder engagement model for the NICCAV project

Engagement with stakeholders critical to HIV treatment and prevention research has a long history [19]. Day *et al.* [18] conducted a systematic review and identified multiple strategies for facilitating stakeholder engagement most of which were to inform protocol development and trial participants' recruitment. There were also few literatures reporting on stakeholder engagement with HIV prevention research conducted in low and middle income countries, countries where stakeholder engagement is considered critically important [20]. Community members themselves identify no justification for omitting community engagement plans during the implementation of research [21]; and if strategically engaged, can actually make critical inputs into decisions on study designs [22]. In view of these, the NICCAV project developed a community stakeholder engagement plan. The community stakeholder engagement programme was contracted to an independent civil society organisation (CSO) that had multiple years of experience working with the community on new HIV prevention technologies by the researchers. This organisation, the New HIV Vaccine and Microbicide Advocacy Society (NHVMAS), developed a community stakeholder engagement plan based on the Good Participatory Practice guidelines (GPP) [14]. Figure 1 shows a pictorial depiction of the community stakeholder engagement model for the study.

Stakeholders engaged include Community representatives working in Jos Metropolis: representatives of support groups for people living with HIV and AIDS in Plateau State, non-governmental organisations, youth, women traditional leaders, Islamic and Christian religious groups. State policy makers and State stakeholders in Plateau State engage include the Ministry of Health and the Plateau State Agency for the Control of AIDS, Network of People Living with HIV (Plateau State chapter), Network of Youths living with HIV (Plateau State Chapter), Network of Civil Society working on HIV and AIDS (Plateau State Chapter, Plateau State Metropolitan Development Board representatives, State Association of Nurses and Midwives, Association of Hospital and Administrative Pharmacists, journalists. At the national level, those engaged were the National Agency for the Control of AIDS, journalists, Civil Society For HIV/AIDS in Nigeria (CISHAN), Network of People Living with HIV, Network of Youths living with HIV & the Network of Civil Society working on HIV and AIDS. The objectives of the community stakeholder engagement research were to (i) constitute a Community Advisory Board (CAB) that had the capacity to effectively engage with the research team; (ii) engage and build the competency of CSOs within the Jos metropolis (the host community of the study) to enable them effectively address the research literacy needs of community members; and (iii) build the capacity of journalists to educate the general public and other stakeholders about HIV vaccine research. The project had specific mid-term (year 1.5) success indicators. Table 1 is a summary of stakeholder engagement plan developed and submitted for ethics approval. We report on the method used for implementing the community stakeholder engagement model, the mid-term evaluation process, and the outcomes of the mid-term evaluation. We then discuss the successes and challenges with implementing the GPP. An end-of-term assessment could not be conducted due to a last quarter cut-back in funding. We therefore cannot objectively discuss the impact of the project.

**Table 1:** Telephone interview guide for the formative evaluation of the NICCAV project

Questions	(1) Excellent	(2) Good	(3) Fairly	(4) Poor	(5) Do not know
**A: Community entry of NICCAV PROJECT:**					
1. Did your participate in the community mapping programme				Yes	No
2. How would you rate the engagement of the community stakeholders in the mapping					
3. How would you rate the relevance of the community mapping was to the NICCAV project					
4. How would you rate the sites selection for the community engagement project					
4a. What specific contributions do you think it had on the NICCAV project					
4b. What suggestions do you have for improving the mapping					
5. How do you rate the community mapping as the community entry strategy of the NICCAV project					
**B: Constitution of NICCAV Community Advisory Board:**					
6. How would you rate the process of selection of the Community Advisory Board?					
7. How would you rate the level of engagement of the Community Advisory Board with research team members					
8. How will you rate the ability of the Community Advisory Board to conduct its duties					
8a. How do you think the Community Advisory Board can improve on its activities.					
**C: Civil Society Society engagement**					
9. How would you rate the process of selection of the Civil Society Organisations engaged on this project					
10. How would you rate the adequacy of the number of Civil Society Organisations engaged on this project					
11. How will you rate your satisfaction with the level of Civil Society Organisations engaged on this project					
12. How will you rate your satisfaction with the research literacy programme conducted by Civil Society Organisations engaged on this project					
12a. Do you have concerns about the research literacy programme and if yes, what is it?					
12b. How do you think Civil Society Organisations can be better engaged on this project?					
**D: Media engagement**					
13. How would you rate the adequacy of the number of journalists engaged on this project					
14. How will you rate your satisfaction with the level of media engaged on this project					
15. How will you rate your satisfaction with the media publicity of this project					
15a. Do you have concerns about the media programme and if yes, what is it?					
15b. How do you think the media programme can be improved for this project?					
**E: Research literacy training and capacity building programme**					
16. Did you participate in research literacy trainings organised for the NICCAV project				Yes	No
17. How would you rate the research literacy guide developed for on this project					
18. How will you rate community research literacy programmes for this project					
19. How will you rate the impact of the research literacy training on your organisation’s ability to engage with research processes					
19a. What specific actions have you taken as a result of the training?					
19b. How do you think the research literacy training can be improved?					
20. Would you like to make suggestions for improving the overall performance of the community engagement programme of the NICCAV PROJECT?					

## Methods

**NICCAV project study design:** the NICCAV study enrolled 534 HIV-1 exposed sero-negative study participants who were 18 years old and above [23] from Jos and its environs, from October 2011 till December 2012 and followed-up each study participants for two years [23].

**Community mapping:** a two-day community mapping was conducted as an entry point into the community with 65 stakeholders engaged with HIV response in Jos metropolis. These were journalists, research team members, representatives of Community Based Organisations (CBO) working in Jos Metropolis, policy makers from the Plateau State Ministry of Health and the Plateau State Agency for the Control of AIDS, Plateau State Chapters of the Network of People Living with HIV, the Network of Youths living with HIV and the Network of Civil Society working on HIV and AIDS, representatives of the Plateau State Metropolitan Development Board, State Association of Nurses and Midwives, Association of Hospital and Administrative Pharmacists, representatives of support groups for people living with HIV and AIDS in Plateau State, non-governmental organisations, youth, women traditional leaders, Islamic and Christian religious groups. The community mapping enabled participants identify (i) populations and groups (socioeconomic and demographics, health status and risk profile, culture and ethnic characteristic) to be engaged on the project; (ii) locations (geographical and neighbourhood boundaries) where potential volunteers can be identified and educational and recruitment efforts can be concentrated; (iii) connections (affiliation, partnership, shared interest, values, motivating forces) between the populations, groups and locations; (iv) stakeholders and leaders (formal and informal leaders, community authorities, and influential persons) to engage. The community map was validated by four CBOs.

**CSO engagement plan:** each of the 20 CBOs actively engaged on the project were expected to reach a minimum of 100 persons per month to discuss the conduct of research in general, HIV prevention, biomedical HIV prevention research and the NICCAV project. Each CBO had a logbook for logging information on participants at their community education programme. This enabled NHVMAS to validate the monthly report on number of participants reached. Each CBO was provided with an educational flip chart specifically developed for the project that served as a research literacy training guide [24]. Training and re-training on use of the flip-chart was conducted during the scheduled monthly meetings. Each CBO was also provided with a field tool-kit that contained samples of the prevention tools shown on the flipchart. During trainings, participants were also shown samples of microbicide study product (specifically the CAPRISA 004 study gel) and Truvada used as a pre-exposure prophylaxis. Coupons for referral of potential study participants were also provided. CBO could invite research team members to their community awareness and education programmes. All the CBO met each month to provide feedback on the monthly activities, challenges encountered and discuss how to resolve the challenges. NHVMAS provided technical support to each CBO on how to integrate the awareness raising and education programmes into ongoing organisational public education. Joint research literacy activities were conducted on HIV Vaccine Awareness day and the World AIDS day. Community programmes were also supplemented with HIV testing activities by CBO with competency to provide HIV counselling and testing.

**Establishment of an Active CAB:** an 11 person CAB was constituted consisting of representatives of the youths, religious groups, the local council, traditional leaders, the media, as well as the health, justice, welfare and women groups based on a decision the decision reached by stakeholders at the community mapping exercise. Organisations were contacted to nominate members to the CAB. The CAB played an advisory role to the project and their activities were guided by a charter and a standard operating procedure developed by the CAB members with support of NHVMAS. CAB members met every month. Research team members participated in the monthly meeting to provide updates on the study progress including the recruitment and retention figures. Twenty-one meetings were held during the lifetime of the project.

**Media engagement programme:** a specific media engagement programme was developed for the project. The objective of the media engagement programme was to increase public awareness about the project. Fourteen journalists selected by the State Chapter of the Union of Journalists were engaged on this project. Journalists had access to study team members and stakeholders when developing stories. For the project, three television stations, two radio stations, four print newspapers and the news agency of Nigeria were actively engaged. Media broadcasts and publications about the study were monitored.

**Building capacity to conduct community awareness and education:** a structured capacity building programme was developed. Every three months for 24 months, 35 CSOs (20 actively engaged on the project and 15 as invited stakeholders), 11 CAB members and the 14 journalists engaged for the project took a two days training on the science of biomedical HIV prevention research using a participatory approach. The topics discussed during the trainings range from basic definition of science terminology to understanding research process, topics on bioethics, protocol review process, informed consent procedures, risk reduction interventions for HIV prevention research, study participants recruitment and retention planning and planning and conducting community education and outreaches. Participants had a tour of the research facility and shown how study participants were consented and enrolled in the study, and how bio-specimens were collected, tested, and stored.

Advocacy programme: NHVMAS developed an advocacy agenda to enable the project engage with community leaders (formal and informal leaders, community authorities, influential leaders and those most likely to be affected by the study). NHVMAS and a research team representative met every quarter with the Commissioner of Health, the Director of the State Agency for the Control of AIDS, the State Coordinator of the Network of People Living with HIV and AIDS and the State Coordinator of the CSOs working on HIV and AIDS to provide updates on the NICCAV project. Stakeholders reached during the quarterly advocacy visits are listed in Figure 1. Thirteen advocacy visits were conducted. Biannual round table meetings were organised for national stakeholders to discuss the NICCAV project. Those engaged included journalists, programmers working in the field of HIV research, those in the development sector, undergraduate students, academia, researchers, drug regulatory officers, policy makers, community advocates, ethicists and members of ethics board. Five round table meetings were organised in Lagos, Jos and Abuja.

**Monitoring and evaluation plan:** the community engagement programme commenced one year after the initiation of the study. Prior to the first and second quarterly education programme conducted for those engaged for the project, a pre-test was conducted to measure basic understanding about research in general and HIV vaccine research specifically. A post-test was conducted at the end of the two days training workshop. These measures were used to assess the impact of the education. One and a half years into the project, a mid-term evaluation of the stakeholders’ engagement programme was conducted. The formative evaluation was limited to the stakeholders that had been reached directly by the project. The evaluation used both qualitative and quantitative assessment to identify how well the project was performing. The evaluation process included a desk review of all project reports and a telephone interview to administer a questionnaire that assessed level of satisfaction with the programmes using a five point likert-like scale (Table 1). The questionnaire was pilot tested for content clarity. The 25 survey participants (10 CBO representatives, four CAB members, seven journalists and four NICCAV project staff) were randomly selected by balloting, from the lists provided by NHVMAS. An external evaluator conducted the evaluation. The proposed end-of-term evaluation planned for the project could not be conducted due to cut-back of the grant three months to the expiration of the project.

**Data analysis:** a descriptive analysis was conducted for the quantitative data. The likert-like scores were re-categorised into three. Scores 1 (excellent) and 2 (good) where re-categorised and coded as satisfactory, and scores 3 (fair) and 4 (poor) were categorised as unsatisfactory. Score 5 remained as unknown. An inductive thematic analysis was conducted to identify salient themes after reading and re-reading the hand written notes from the telephone interviews. The findings were triangulated with the findings from the quantitative data.

**Ethical consideration:** the main NICCAV research study received ethical approvals for the study [12]. NHVMAS obtained ethics approval for its stakeholders' engagement programme from the Plateau State Specialist Hospital, Jos (NHREC/05/01/2010B). All data were handled confidentially. Verbal consent was obtained from all participants during the mid-term evaluation.

## Results

**Level of attainment of project's good participatory practice strategies:** the study built the capacity of 35 CSOs; successfully engaged 16 (four CBOs disengaged with the project prior to the evaluation) rather than two CSOs (800% above target) to conduct research literacy training; and reached 24,882 rather than 2,000 community members (1,244.1% above target). The number of persons reached represents 3.03% of the 821,618 adults resident in Jos metropolis [25]. Also, an 11 rather than a 15 man CAB (in line with the decision of stakeholders) held monthly meetings and engaged with the project staff for feedbacks and discussions on the research study.

**Capacity building programme:** all respondents adjudged the capacity building programme empowering. The two pre and post tests conducted for stakeholders (CSOs, CAB members, journalists, policy makers) showed significant difference in the pre- and post-tests scores (50.03 +16.86 vs 59.79 +15.79; p=0.004).

**Community entry, awareness and education programme:** all the survey respondents participated in community mapping. They found the mapping appropriate and relevant, and acknowledged that CBO were effectively engaged throughout the project. They were satisfied with the community education and awareness programme. However, only 40% of respondents were satisfied with the level of financial support provided for the community awareness and education programme. The CBO noted that the success of the NICCAV community education and awareness programme was dependent on their ability to integrate the programme into existing organization programmes. This led to four of the original 20 CSOs not been able to continue with the project and some CSOs not been able to meet their monthly targets.

Only 70% of respondents felt that the community awareness and education coverage was satisfactory and 60% felt satisfied with the site selection for the project. Participants who were not satisfied with site selection and the community awareness and education coverage all related issues that had to do with security concerns. At the time of conduct of the study, Jos was involved in a major religious crisis, which tore the town into the Muslim and the Christian quarters. The research clinic was situated in the Christian part of the town. This was a major challenge for referring clients who reside in the Muslim areas as some community members perceived the project to be a Christian programme since it was located in the Christian quarters. This perceived religious affiliation of the study made it challenging to conduct community awareness and education programmes for the communities in the Muslim quarters. Participants suggested that research out-station clinics should have been located in the Muslim quarters to facilitate access of Muslims to the project.

**Engagement with the media:** all the respondents were satisfied with the media engagement and commended the active engagement of journalists throughout the lifecycle of the project. Respondents acknowledged that the level of media engagement on this project was the first for any research project conducted in the country. The project recorded 12 radio and television broadcasts and 10 media publications. The total population reached by these media houses was estimated to be 40 million (1.7 million people in Plateau State and 38.3 million people outside Plateau State). Table 2 is a list of some of newspaper publications and electronic messages about the study. Despite the enthusiasm on engagement of the media, the limited budget allocated for the media engagement programme also meant journalists had to look for free slots to discuss the NICCAV project rather than create an active ongoing programme that specifically focused on the NICCAV project.

**Table 2:** Some media publication on the NICCAV project

Name of Media house	Title of publication	Linkage
Leadership	Nigeria joins race search HIV vaccine	http://leadership.ng/nga/articles/35369/2012/09/21/nigeria_joins_race_search_hiv_vaccine.html
nigeriarising.com.ng	Nigeria-to-develop-local-hivaids-vaccine	http://nigeriarising.com.ng/sector-report/health/nigeria-to-develop-local-hivaids-vaccine/
BusinessDay	Nigeria joins search for HIV vaccine	http://newsbreaknigeria.com/news/Nigeria+joins+search+for+HIV+vaccine+-+BusinessDay
The Guardian	Search begins for-Nigeria’s-own-HIVAIDS-vaccines	http://www.ngrguardiannews.com/index.php?option=com_content&id=98684:search-begins-for-nigerias-own-hivaids-vaccines
News Agency of Nigeria	Nigeria joins the race for the search for HIV vaccine	http://www.nanngronline.com/section/healthgender/nigeria-joins-the-race-for-the-search-for-hiv-vaccine
Premium times	Nigerian researchers seek cure for HIV	http://premiumtimesng.com/news/100960-nigerian-researchers-seek-cure-for-hiv.html
Nigeria Health forum	Federal government out tackle HIV/AIDS Nigeria	http://www.nigeriahealthforum.com/health/f24/fg-out-tackle-hiv-aids-nigeria-2306/

## Discussion

This community engagement project developed a stakeholder engagement plan, a stakeholder advisory mechanism, a communication plan and a stakeholder education plan in line with requirements of the Good Participatory Practice (GPP) guidelines, a formal document prescribing community engagement principles for biomedical HIV prevention research. The community mapping enabled the study to understand the profile of the study site in line with the objective of the formative research stipulated by the GPP. In addition, the study developed an advocacy plan which helped to facilitate its engagement with strategic political stakeholders beyond the study site. The advocacy plan went beyond the stipulations of the GPP. The implementation of the community engagement plan enabled the project to build the knowledge and skills of the primary contacts to conduct research literacy programmes for the Jos community. The implementation of these plans were adjudge satisfactory. The community stakeholder engagement model employed for the NICCAV study was participatory and provided a platform for stakeholders to actively engage in the design of the stakeholder engagement progamme thereby giving credibility and legitimacy to the community engagement process and probably accounts for the high level of satisfaction by respondents engaged in the programme evaluation. It was less of a researcher driven top-down approach that otherwise could not have fostered meaningful partnerships and continuous dialogue [18]. The engagement of an independent institution to handle the community engagement programme enhanced the ability of the community engagement structure and systems developed for the research study to operate autonomously and independent of influence of the research team.

However, the low budgetary allocations limited community coverage with research literacy information. The inappropriate budgeting was a result of developing a community engagement plan after the protocol approval by the ethics committee and sponsors. Stakeholder engagement is often conducted using researcher driven, top-down methods. This often involves formal social science methods such as in-depth interviews or focus group discussions. It is unclear how effective these methods are for fostering meaningful partnerships and continuous dialogue as the GPP guidelines recommend [3]. Additionally, the extent to which top-down engagement methods can inform the design and conduct of HIV clinical trials depends entirely on trial researchers. Thus, while the GPP guidelines recommend that trial researchers carefully consider and select from the range of possible advisory mechanisms [3]. We felt that the media engagement programme was also a strong component of this model. Medeossi *et al.* [26] highlighted the importance and benefits of using multiple media outlets to engage the community for biomedical HIV prevention research. Although we could not demonstrate the impact of the media engagement programme, the active dissemination of information about the project was a crucial step in creating awareness and generating interest about the project. The study had its limitations. First, it could not develop an issue management plan as prescribed by the GPP. The issue management plan should describe how research teams intend to manage any unexpected developments that may emerge before, during, or after the trial [18]. While the community engagement project recognised the need for this, the project was unable to develop these plans as it required the active engagement of the research team. Attempts made to develop this plan failed. We felt this was because the attempt was made by an organisation external to the research team. Though engaging an independent organisation to manage the community engagement process is commendable, this made it challenging to actively engage the research team in the development of this plan. The autonomy of the community engagement program however, came with its advantages. It enabled the project develop a sustainable community engagement programme rather than focusing on promoting study participants recruitment and retention [27-29].

Community members were also able to identify their need for the research [30], helped the project identify ways of minimising associated internal and external risks [31], and built research literacy competency of the community stakeholders. It also facilitated the constitution of a CAB whose members' allegiance was to their primary constituencies thereby addressing some of the concerns research projects had about CABs [32]. Secondly, the stakeholder engagement programme commenced after study participants recruitment had commenced. This is not in line with the GPP requirement: community stakeholder engagement programs should commence at conceptualisation of the research question design of the study [18,33] and continue throughout the research life-cycle [33]. The failure to engage stakeholders early in the NICCAV project's life-cycle made the project fail to design a study that was sensitive to the religious inclination of the community. Although the study met its recruitment target [19], the likelihood was that the distribution of the recruited study participants' may have shown religious bias. Third, the limited financial support for the project came with challenges. The community engagement program's budget was cut back to 37.5% of its initial budget because of cut-backs in grant allocation by sponsors. Yet, the scope of the program could not be reduced and the project engaged 14 extra CBO. Although the programme leveraged on many existing project to conduct its activities [34], this approach limited the extent to which the community research literacy and the media engagement programmes was executed. Prior programs had identified the constant threat to the funds allocated for community engagement when the research project faces financial constraints [35,36]. We recommend that future community stakeholder engagement programme budget appropriately for its programmes. Despite these limitations, this study showed how studies can innovatively achieve its goals through engagement of local, state and national political stakeholders' in a research project in line with the recommendations of the GPP.

## Conclusion

The NICCAV study community stakeholder engagement model was an effective model for implementing most of the GPP guidelines. It was however, unable to engage the research team to develop an issue management plan. It highlighted the limitation with not engaging communities at the project conceptualisation stage and the challenges with inadequate budgetary allocations for its community stakeholder engagement programme. The development of an advocacy and monitoring plan strengthened the process of engaging political stakeholders and capturing information on project outcomes. These should be considered components of the GPP.

### What is known about this topic

- Vast literature on the need for community engagement for biomedical HIV prevention research;
- Guidelines on how to conduct community engagement in biomedical HIV prevention research,
- Very sparse evidence on the process, outcomes and impact of community engagement in biomedical HIV prevention research.

### What this study adds

- This study information on the 'how' of community engagement in research;
- This is a report of an indigenous process of facilitated community engagement with a biomedical HIV prevention research howbeit a demonstration project;
- It also provides evidence on the outcomes of a community engagement programmes based on monitoring and evaluation indicators put in place at the designing of the project, and adds unique information to the field of GPP implementation.

## Competing interests

The authors declare no competing interests.
